# Nurses’ knowledge and attitudes towards palliative care and death: a learning intervention

**DOI:** 10.1186/s12904-021-00738-x

**Published:** 2021-03-25

**Authors:** Yanping Hao, Lixuan Zhan, Meiling Huang, Xianying Cui, Ying Zhou, En Xu

**Affiliations:** 1 College of Nursing and Department of Neurology of the Second Affiliated Hospital of Guangzhou Medical University, 250 Changgang Dong RD, Guangzhou, 510260 People’s Republic of China; 2 Nursing Department of the Third Affiliated Hospital of Guangzhou Medical University, Guangzhou, 510150 People’s Republic of China; 3 Nursing Department of Hospital of Traditional Chinese Medicine in Yuexiu District, Guangzhou, 510030 People’s Republic of China

**Keywords:** Palliative care, Nursing, Internet-based intervention, Knowledge, Attitude

## Abstract

**Background:**

In many countries, nurses are ill-prepared to provide care to patients with terminal illnesses. Limited education and training affect their ability to deliver proper palliative care. Only a few studies have explored appropriate and effective training methods of palliative care in China. Therefore, we aimed to provide evidence for a palliative care training system by appraising the effects of a mixed-method intervention on participants’ knowledge of palliative care and attitudes towards dying patients and death.

**Methods:**

An e-learning intervention approach was adopted for 97 nurses from oncology departments across five hospitals, using a mobile terminal combined with a virtual forum and face-to-face interactions. We conducted a pre- and post-training evaluation through the Palliative Care Quiz of Nursing (PCQN), Frommelt Attitude Toward Care of the Dying Scale Form B (FATCOD-B), and Death Attitude Profile-Revised (DAP-R).

**Results:**

After a three-week intervention, there was a significant increase in the PCQN and FATCOD-B scores as compared to the baseline. For PCQN, the total score increased from 10.3 ± 1.9 to 11.1 ± 2.2 (*p* = .011) and the score for management of pain and other symptoms increased from 7.7 ± 1.7 to 8.4 ± 1.7 (*p* = .003). FATCOD-B scores increased noticeably from 100.6 ± 7.9 to 102.9 ± 8.9 (*p* = .019). The DAP-R scores showed no obvious difference between pre- and post-intervention results.

**Conclusions:**

The mixed-method intervention was effective in improving participants’ knowledge and attitudes about palliative care. The implementation of training for nurses at appropriate intervals during both education and professional life is required, especially regarding the improvement in participants’ attitudes towards death. Therefore, palliative care training in China should receive more attention.

## Background

Global population ageing and the rising prominence of chronic non-communicable diseases have resulted in an increased demand for palliative care nursing [1]. With the proportion of patients with incurable diseases increasing each day, [2] the need for high-quality palliative care has also consequently increased challenges for healthcare educators [3, 4]. In 2017, the National Health Commission of the People’s Republic of China released basic standards, management specifications, and practice guidelines for palliative care centres. In addition, a pilot programme of palliative care was conducted in some hospitals in China [5]. However, access to palliative care, patients’ rights, and patient referral criteria have not been defined or clearly specified. The implementation of palliative care in China is widely restricted by limited availability of services, shortage of skilled staff, and the lack of appropriate training systems [6, 7]. Many patients suffering from life-threatening illnesses are struggling with poor health conditions due to unmet care needs towards the end of their lives [6, 8]. Providing professionally trained palliative care staff is a potential solution for improving the quality of life of patients facing life-threatening illnesses.

The palliative care competence of nurses is a strong contributor to improving the outcomes of high-quality care [9]. However, Chinese nurses face barriers in their clinical competencies as they do not receive adequate palliative care education in nursing schools, [10, 11] and associated professional training in this field at their workplaces [12, 13]. Accordingly, educational training is a crucial element for enhancing nurses’ competence [14]. To eliminate this educational gap, establishment of a professional training system in palliative care for nurses in China is imperative.

Several key factors are associated with the competence of nurses in palliative care, including their knowledge and attitudes towards palliative care and death [15, 16]. Previous studies indicated that educational training for healthcare professionals had a positive influence on their confidence, knowledge of palliative care, and attitudes towards caring for dying patients and death [17–20].

Traditional face-to-face training is increasingly being replaced by online education. Electronic learning (e-learning) is a feasible and acceptable approach towards the delivery of high-quality education. The effectiveness of online training in palliative care has mainly been demonstrated through computer-assisted learning [21, 22]. Few studies have reported on these educational programmes, particularly by mobile terminals. Because the location of learning is no longer restricted to a fixed place, it is easy to take the course at any given location.

A professional training system of palliative care is urgently needed in China and evaluating the effectiveness of this system will assemble reliable evidence and quality scientific studies to move forward. In this study, we aimed to provide evidence for this future system by investigating the training needs of palliative care nurses and customising an educational intervention according to their feedback. We implemented the intervention based on e-learning via a mobile terminal, combined with a virtual discussion forum and face-to-face interactions, which was followed by evaluating the training effect on nurses’ knowledge of palliative care and their attitudes towards caring for dying patients and death.

## Methods

### Study design

This study employed a non-randomised, single-arm design to explore the effect of an intervention programme, for nurses, on the knowledge of palliative care and attitudes towards caring for dying patients and death. Questionnaires were distributed among participating nurses from five hospitals.

### Settings and participants

From September to December 2018, we conducted the study across five hospitals: The Second Affiliated Hospital of Guangzhou Medical University, The Third Affiliated Hospital of Guangzhou Medical University, Community Health Service Centre in Hongshan Street, People’ Hospital in Liwan District, and Hospital of Traditional Chinese Medicine in Yuexiu District, Guangzhou, China. All participants were registered nurses, aged 18 to 60 years. We assumed that in this research, the probability of a type-I error was 0.05, the probability of a type-II error was 0.1, and the allowable error δ was 0.3 times the standard deviation, which was taken from *Advanced Medical Statistics* [23]. The standard deviation (SD = 0.31) of the sample size calculation was quoted from previous study [24]. Therefore, the allowance error was equal to 0.3SD (δ = 0.093). Based on these statistical data, the minimum sample size was 96 by a formula of sample size calculation, plus 10% of sample loss, and the estimated sample size should be 106 cases. Actually, 125 nurses from the oncology departments of these hospitals were screened for eligibility and 109 of them were enrolled.

### Measurements

The demographic characteristics collected from the participants were: gender, age, education level, received school education in palliative care, work experience, training experience in palliative care before the intervention, and training needs for palliative care. The major outcomes of the study were measured by the Palliative Care Quiz of Nursing (PCQN), [25] the Frommelt Attitude Toward Care of the Dying Scale, Form B (FATCOD-B), [24] and the Death Attitude Profile-Revised (DAP-R) [26]. All scales had confirmed reliability and validity [27–30].

The PCQN covers three categories in end-of-life care: (i) philosophy and principles of palliative care (4 items), (ii) management of pain and other symptoms (13 items), and (iii) psychosocial aspects of care (3 items). Total scores ranged from 0 to 20, with higher scores indicating better knowledge.

The FATCOD-B comprised 30 items. The scores ranged from 30 to 150, with higher scores indicating a more positive attitude towards care for the dying.

The DAP-R, a 32-item scale comprised five subscales: fear of death (7 items), death avoidance (5 items), neutral acceptance (5 items), escape acceptance (5 items), and approach acceptance (10 items). Each subscale, with scores ranging from 1 to 7, represented one of the five attitudes towards death and a higher score indicated a greater tendency to share the corresponding attitude towards death.

### Procedure

At the beginning of the research, the participants were provided a thorough explanation of the study and notified that the participation was voluntary, and they could withdraw from the study at any time. All questionnaires were anonymous. The participants were informed about data confidentiality and signed informed consent forms. The self-administered, written questionnaire was delivered to each participant to complete at two time-points: pre-intervention and post-intervention. The first phase of the survey involved participants’ demographic characteristics, training requirements for palliative care, and assessment of their knowledge of palliative care and attitudes towards caring for dying patients and death. The training course, as an intervention, was customised according to the information collected from participants regarding training requirements and implemented over a three-week period. Participants were then given the PCQN, FATCOD-B, and DAP-R questionnaires for the second phase of the survey immediately upon completion of the intervention. Finally, baseline data were compared with post-intervention data to evaluate the effects of training on nurses’ knowledge of palliative care and their attitudes towards caring for the dying patients and death. The Ethics Committee of Guangzhou Medical University (L180920) approved the study protocol.

### Intervention

The training content of the educational intervention was designed based on the data collected on participants’ training requirements for palliative care. It consisted of three modules: life and death education, bereavement, and specialised nursing in palliative care. The courses in these modules were video-recorded and delivered by a professor who has specialised in life and death education for many years. Participants completed 45 video segments in the three modules. They could access the educational material with ease, free of charge, and could repeatedly play each video segment via the We-chat application on their mobile phones or tablets. They could complete the learning tasks in their own time and environment via Wi-Fi or a 4G mobile communication network.

One researcher acted as the administrator and principal investigator of the study, designing the learning progress plan (course segments of Module 1 were asked to be completed in two weeks and Modules 2 and 3 to be completed in one week) and following the status of learning through both virtual and face-to-face interactions. For virtual interactions, the researcher established a forum via We-chat ensuring that all participants joined through their mobile phones. During this interaction, all topics related to the training programme could be discussed by the participants by sending instant messages during their learning experience.

For face-to-face interactions, the researcher held weekly meetings with participants in a hospital conference room. The researcher reviewed the status of their learning and helped participants understand the importance of the course. The participants could freely share their feedback with each other. The training lasted for three weeks (21 ± 1 day) under the supervision of head nurses in participating hospitals who had been instructed on the training plan prior to the commencement of the study. Additional details about the course segments of each module are shown in Table 1.

**Table 1:** Intervention programme modules

Module	Video segment
Module 1: Life & Death	**1**^**st**^ **week**
• Life	1. Origin of life (15’14”)
	2. Where does the human being come from (12’54”)
	3. Thinking about an individual life (9’35”)
	4. Living in the moment (9’9”)
	5. Diversity of lives (3’34”)
	6. Meaning of life (12’8”)
• Death	7. Nature of death (10’42”)
	8. Death is someone else’s business (8’2”)
	9. Being towards death (11’19”)
	10. Fear of death (11’5”)
	11. Surviving beyond death (12’33”)
	12. Meaning of death (11’57”)
• Death with Dignity	**2**^**nd**^ **week**
	13. What is dignity? (9’57”)
	14. Could death be achieved with dignity (10’30”)
	15. Understanding death with dignity (9’8”)
	16. Necessity of death with dignity (8’29”)
	17. Influencing factors of death with dignity (8’45”)
	18. Enlightenment of death with dignity (3’29”)
• Dying	19. What is dying? (14’11”)
	20. What is the dying experience? (8’33”)
	21. Kubler Roth’s view of the dying experience (12’10”)
	22. Moody’s research on the dying experience (7’17”)
	23. Research status on the dying experience (9’17”)
	24. Cases of the dying experience (18’27”)
	25. Non-soul theory about the dying experience (3’46”)
• Death Education	26. Death culture and the education of life and death (30 ‘24”)
	27. What is death education? (8’29”)
	28. Aim of death education (9’33”)
	29. Theme of death education (9’16”)
	30. Development process of death education (5’22”)
	31. Practice of death education (6’22”)
Module 2: Palliative Care Nursing	**3**^**rd**^ **week**
	32. Concept of palliative care^a^ (8’32”)
	33. Philosophy of palliative care (6’29”)
	34. Development process of palliative care (7’59”)
	35. Target population of palliative care (10’14”)
	36. Importance of palliative care (6’50”)
	37. Theoretical basis of palliative care (7’40”)
	38. Pain relief and palliative care (7’52”)
	39. Meaning of palliative care (4’44”)
Module 3: Counselling for Bereavement & Grief	40. What is bereavement and grief counselling? (10’30”)
	41. Necessity of counselling for bereavement and grief (5’29”)
	42. Aim of counselling for bereavement and grief (6’45”)
	43. Practice of counselling for bereavement and grief (6’34”)
	44. Principle of counselling for bereavement and grief (10’38”)
	45. Contents and methods of counselling for bereavement and grief (9’19”)

^a^Concept of palliative care refers to the WHO definition of palliative care [31]

### Statistical analysis

SPSS 25.0 (SPSS, Inc., Chicago, IL, USA) was used for statistical analyses in this study. For all statistical procedures, *p* < .05 was deemed significant. Categorical variables were expressed as numbers and percentages. The effect of the educational programme was determined by comparing the PCQN, FATCOD-B, and DAP-R post-intervention scores with the pre-intervention scores. Differences were calculated and analysed with paired-samples *t*-tests.

## Results

**Fig. 1:**
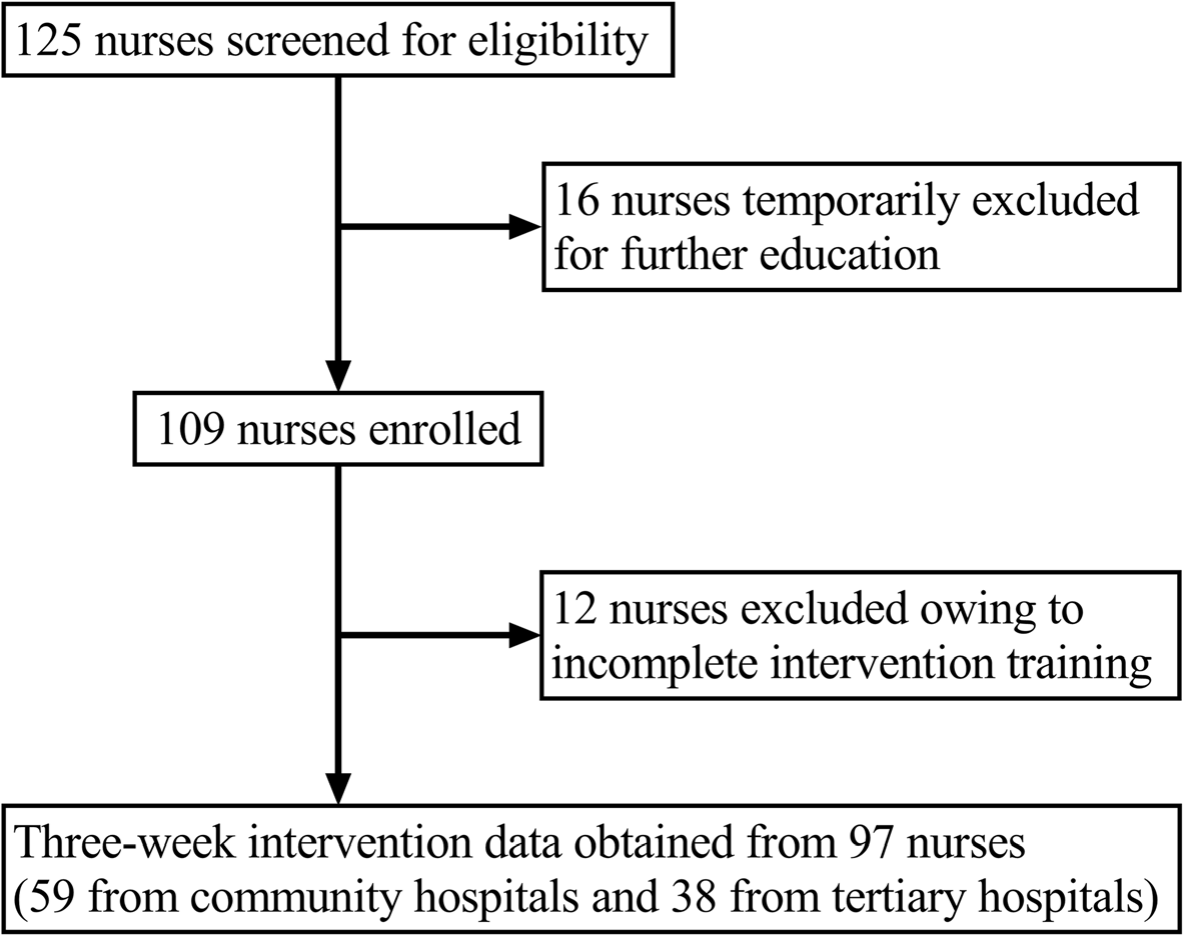
Participants’ flow (*N* = 97)

Initially, 125 candidates were eligible and 16 nurses were excluded for their personal further education. Then 109 candidates representing five hospitals attended the educational intervention programme and took the survey promptly after completing their training. Ninety-seven participants gave an adequate number of responses for the analyses to be conducted (89%). Participant flow is shown in Fig. 1.

### Participants’ characteristics

Participants’ characteristics are shown in Table 2. Most of the participants were women, aged 18 to 30 years. Most participants had five or fewer years of experience. Approximately two-thirds of the participants had not previously received any palliative care training. Almost all reported that they required palliative care training.

**Table 2:** Demographic characteristics of participating nurses

Variable	n	%
Gender		
female	94	96.9
Age (years)		
18–30	72	74.2
31–40	18	18.6
41–50	7	7.2
Educational status		
associate’s degree (3 years)	12	12.4
bachelor’s degree	37	38.1
master’s degree or above	48	49.5
Work experience (years)		
≤2	16	16.5
3–5	49	50.5
6–9	13	13.4
≥10	19	19.6
Training needs for palliative care		
no	3	3.1
yes	94	96.9
Training experience in palliative care		
no	58	59.8
yes	39	40.2

### Main outcomes

**Fig. 2:**
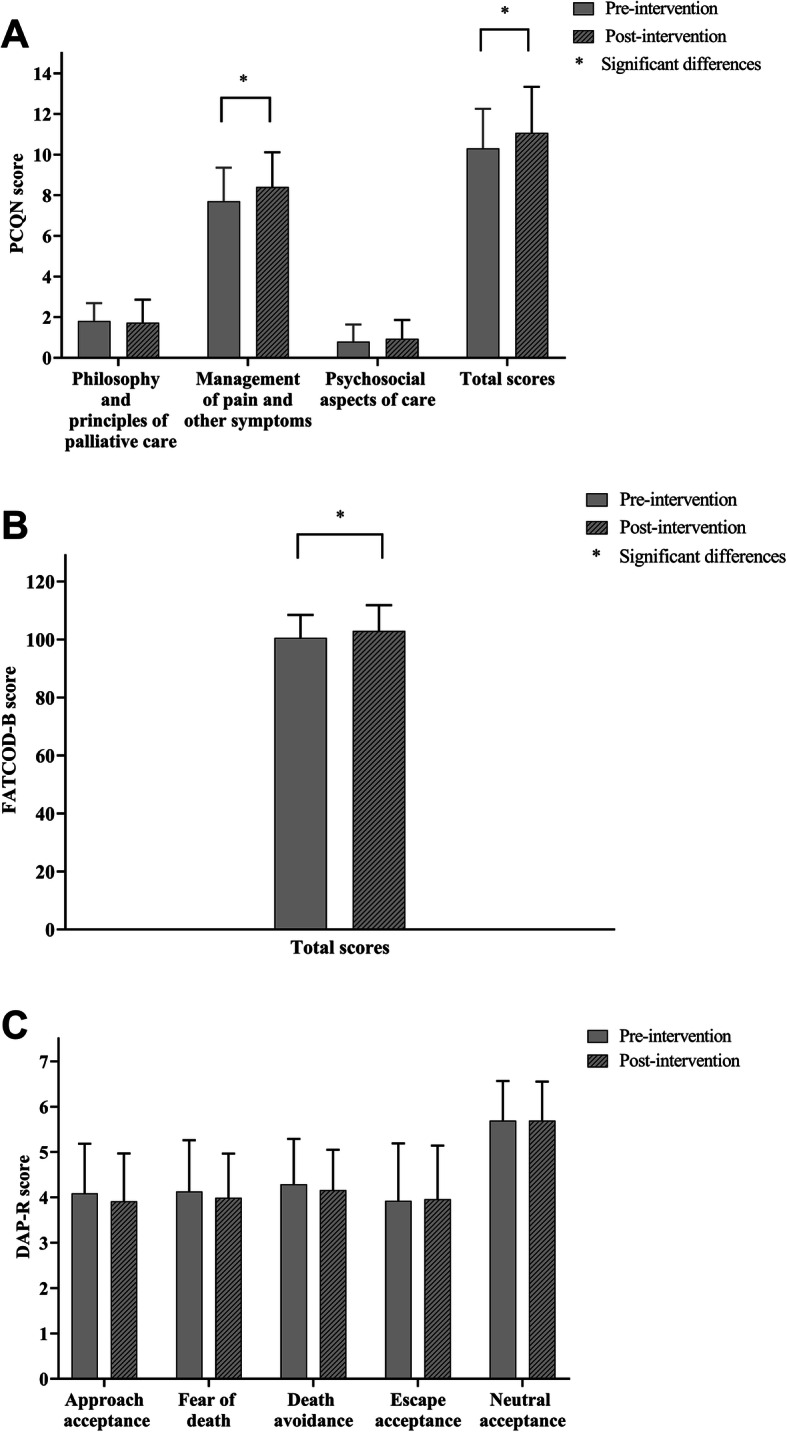
PCQN, FATCOD-B, and DAP-R scores pre- and post-intervention (*N* = 97). **a**, Total PCQN scores ranged from 0 to 20, with higher scores indicating greater knowledge. Scores in philosophy and principles of palliative care ranged from 0 to 4, scores in management of pain and other symptoms ranged between 0 and 13, and scores in psychosocial aspects of care ranged from 0 to 3. PCQN: Palliative Care Quiz of Nursing. **b**, Scores on the scale ranged from 0 to 150 with higher scores indicating a favourable attitude towards care for the dying. FATCOD-B: Frommelt Attitudes Towards Care of the Dying Scale Form B. **c**, Scores on each subscale, ranging from 1 to 7, represented the five attitudes towards death. A higher score indicated a greater tendency to share the corresponding attitude towards death. DAP-R: Death Attitude Profile-Revised. Data were presented as estimated means and deviations. The height of the error bar in Fig. 2 is equal to the mean value of the variable plus the standard deviation. Paired *t*-tests were used for analysis of the difference between pre- and post-intervention scores. *p ≤ .05 for differences between pre- and post-intervention

After participation in the educational programme, significant changes in PCQN and FATCOD-B scores were noted. The total score of PCQN changed significantly from pre-intervention (10.3 ± 1.9) to post-intervention (11.1 ± 2.2; *p* = .011). Management of pain and other symptoms in PCQN increased markedly from 7.7 ± 1.7 to 8.4 ± 1.7 (*p* = .003). The FATCOD-B pre-intervention score of 100.6 ± 7.9 increased to a post-intervention score of 102.9 ± 8.9 (*p* = .019). Through a self-matching test, the DAP-R score showed no obvious difference between pre- and post-intervention (*p* > .05; Fig. 2).

## Discussion

### Main findings

This study identified that the knowledge of palliative care of participants could be improved significantly by an educational intervention, which is also consistent with prior findings [18, 19]. It was found that the nurses’ attitudes towards caring for dying patients also significantly improved after the intervention programme. This is also in line with earlier studies that examined educational interventions for palliative care nurses [18, 29].

However, our study could not demonstrate any significant changes in participants’ pre- and post-intervention attitudes towards death. Some studies reported a significant effect of interventions tackling this issue in other countries [32, 33]. The DAP-R subscale mean scores for fear of death and approach acceptance significantly increased at the end of training in a prior study [33]. The duration of the intervention in our study may not have been sufficient to change nurses’ attitudes towards death. Additionally, cultural values, ethical standards, and religion may differ in China compared to other countries, where studies have shown changes in nurses’ attitudes towards death after interventions [34, 35]. Talking about death is considered a taboo in China, like several other Asian cultures [32, 36]. In different Asian cities, nurses’ attitudes towards death may also be influenced by the social, cultural, and organisational circumstances of practice [34]. We recommend that further studies on palliative care education be conducted in China.

### Strengths and limitations of the study

To reduce sample bias, participants were recruited from the oncology departments of five hospitals, and not just from a single institution [32, 37, 38]. This ensured sampling diversity and representativeness, as well as provided sufficient data for our study. All participants were engaged in caring for dying patients as part of their daily routine work.

Our study applied a mobile-based e-learning approach instead of the web-based method used in other studies, [39] which ensured participants learned with more flexibility and portability without time and location restrictions. Moreover, sending instant messages through the forum enabled real-time communication. Weekly face-to-face Q&A interactions were established to help resolve any learning issues, which made communication more direct and efficient. The integration of multiple methods adopted into the educational programme allowed learning to become more united, convenient, and economical.

There were several limitations to this study, including a relatively small sample size. To verify the effects of the intervention programme, a control group needed to be established [32]. It is difficult to establish a control group across multiple hospitals and ensure the baselines of the control group match those of the experimental one. Further, this study only examined the immediate effects of the educational intervention. Ideally, follow-up studies with the same participants could determine its continued effects on nurses’ knowledge and attitudes towards palliative care and care delivery [17]. Additionally, the duration of the intervention was relatively short. The effectiveness of an intervention in participants’ attitudes towards death may require highly integrated design courses. When it comes to the training contents, an interdisciplinary collaboration approach, which is essential for healthcare providers in clinical practice, was not involved in this study. Furthermore, clinical practice in palliative care in China is not well-developed, and there is a shortage of qualified healthcare providers, who play an important role in ensuring high-quality education delivery [40].

### What this study adds

It is worth noting that the strong demand of participants for palliative care training reflected the current deficit of existing professional training in caring for dying patients. Therefore, a curriculum provision of palliative care education in medical colleges and implementation of workplace training are strongly recommended as quickly as possible, in China. Undoubtedly, this initiative has the potential to guide the creation of a more professional educational training system to eliminate the gap between the training requirements and the provision of palliative care education.

This study showed that a combined-method educational strategy was effective and feasible. It considered how to deploy the available resources to organise and choose a suitable training method that was no longer restricted by factors such as time, space, and finances. It also indicated that a pre-survey based on the training needs of participants could ensure that the training courses are effectively designed and delivered to meet the expectations. The educational strategy developed in this study may be worth developing and disseminating in future initiatives.

Moreover, this study recommended that unified and professional-access criteria be established to form an authorised and recognised qualification system in palliative care in China. In other words, the competence of palliative care staff could be specified and professional standards of staff training could be clarified, which would give access to more palliative care staff to attain the required qualification in this domain. Evidently, the implementation of training for nurses at appropriate intervals during both the educational period and professional life is required. Accordingly, the palliative care curriculum design needs to be gradually improved, and education providers’ competencies need to be further enhanced.

## Conclusions

In summary, our findings provided convincing evidence that mobile terminal-based e-learning combined with virtual and face-to-face interaction was an effective approach to educational training. We confirmed that educational training could exert a positive impact on trainee nurses, enhancing their knowledge of palliative care and improving their attitudes towards caring for the dying patients. This study provided scientific and promising evidence for future education and training systems in palliative care.

## Data Availability

The datasets used and/or analysed during the current study are available from the corresponding author on reasonable request.

## Abbreviations

PCQN: Palliative Care Quiz of Nursing
FATCOD-B: Frommelt Attitude Toward Care of the Dying Scale, Form B
DAP-R: Death Attitude Profile-Revised
